# Disease of the Mucous Membrane of the Mouth and Alimentary Canal a Cause of Disease of the Teeth, Gums and Alveolar Processes

**Published:** 1856-07

**Authors:** 


AMERICAN JOURNAL

OF

DENTAL SCIENCE.

Vol. VI. NEW SERIES—JULY, 1856. No. 3.

### ORIGINAL COMMUNICATIONS.

### ARTICLE I.

*Disease of the Mucous Membrane of the Mouth and Alimentary Canal a Cause of Disease of the Teeth, Gums and Alveolar Processes.* By A. A. B.

All morbid influences acting upon the tissues of the mouth effect an alteration in the various secretions which lubricate the teeth, constituting a fearful, and the chief, cause of their destruction.

It becomes most important to know the influences producing such effects, and to have a thorough comprehension of the pathological changes of the general system, as well as of the local parts, to enable the practitioner to administer suitable remedies. Believing that these considerations are not generally recognized, and much experience, with continued observation, forcing a conviction of their necessity upon me, I have chosen this subject for a paper in the Journal, trusting that the observations made will elicit further communications from abler pens upon the same topic.

In many diseases we can trace with absolute certainty that their immediate baneful effect is in the mouth and subjacent tissues, evident in parotitis, when, not unfrequently, the entire glandular system of this part is involved, and sometimes becomes a diffused inflammation of the entire mucous membrane.

Again, in tonsillaris, it is difficult to find any solution to the induction of the disease to these glands, except through the agency of the mucous membrane, and to which the curative treatment is principally addressed. Observe, in its grave types, how it creeps to distant parts—the whole of the fauces becoming a sloughing surface, the entire mouth is gradually encroached upon; then the disease finds its way down the esophagus, ulcerating the eustachian tubes to the ears; in fact introducing disease along the whole course of the alimentary canal.

Laryngitis presents equally startling facts, through inflammation of the mucous membrane. It makes its approach with the symptoms common to most of the inflammatory fevers, chilliness, heat, &c., followed by a changeable, indistinct voice, viscid mucous secretions, gradual swelling of the tongue, face and eyes, the fauces red and turgid, implicating the tracheal and bronchial vessels, vitiating their secretions, so as to produce, in some cases, strangulation from its filmy nature.

In bronchitis, we have a much more serious difficulty of this kind, as it is generally experienced in a chronic form, sometimes of extreme duration, lasting through the life of the individual. It is a common error to believe that, in most instances of this disease, the bronchial tubes are alone implicated; the inflammation generally spreads, by means of the mucous membrane, over the whole range of the windpipe, including the larynx and the most intricate ramifications of the bronchia. The gums, and even the glands of the mouth, are most frequently found to partake of the inflammation, changing the nature of the secretions into acrid and vitiating matter, producing caries and erosion of the teeth, and often absorption of the alveoli.

Close observation will, in numberless cases, enable one to detect this result in chronic bronchial irritation; delicate chemical tests, by skillful manipulations, will confirm the opinion beyond all doubt or controversy; and although the mucous secretions, found in the larger parts of the tube, are said to consist entirely of the gluten, or coagulable lymph of the blood, together with slight traces of serum and fibrin, that of the salivary glands will be found, most generally, to have changed its alkaline to an acid character. In the origin of this disease, the lining membrane of the mouth will be found alternately pale and livid, slightly swollen, attended by heat and dryness, which, gradually descending, fixes itself in the trachea, where it results in engorgement, as before mentioned, or in a dry, hacking cough. In the young, it assumes, almost invariably, the acute form, exhibiting itself in catarrhal fever, attended, not unfrequently, by violent spasmodic action, or dry and ineffectual coughing.

Dr. Cullen, treating of peripneumonia and pleurisy, distinctly asserts, that one of the chief causes is the application of cold to the mouth, attended with the general concomitants of inflammation; and although a general idea prevails that persons suffering under any of the forms of phthisis are peculiarly exempt from diseases of the mouth, I have never found it verified by observation. On the contrary, I have frequently witnessed a diseased condition of the mucous membrane of the mouths of patients laboring under pulmonary troubles, indicated by a pale anæmatous condition of the gums; the apices between the teeth are often red and slightly swollen, but not so subject to hemorrhage, or as sensible to mechanical contact as in other morbid conditions. The salivary secretion becomes frothy, and sometimes even glutinous; refuses to mingle with the mucus, somewhat similar to its condition in mania, inebriation and hydrophobia. One patient, particularly, was compelled to cleanse the mouth every few minutes from the sputa collected, which retained, became extremely offensive.

There can be little doubt concerning the intimate relationship existing between the condition of the lungs and the mucous membrane. It is conceded that pulmonary diseases, when hereditary, are strongly indicated by many symptoms, and by none more than by the size, color and consistency of the teeth, the most remarkable appendages of the mucous membrane, and that their texture and health, under these circumstances, must be changed, is an unavoidable conclusion. A further proof of this fact is fully presented in the history of a patient, who was subject to frequent attacks of pneumonia; during such times as the disease was present, the fauces, uvula, and soft palate became injected with blood, so as to be covered with patches of a deep red color; the little capillaries were completely congested, radiating from and interlacing the edges of these patches, giving a painful soreness to the parts and much inconvenience in swallowing. Before the age of thirty, the teeth had all been sacrificed, from the acidulated condition of the juices of the mouth. After their removal, the same phenomena supervened, the gums becoming sore and swollen as before, and again returning to a healthy condition upon the cessation of the cause originating the disturbance.

In many cases of asthma, we cannot perceive any other trouble than an irritated condition of the mucous membrane lining the mouth, bronchia, &c.; although the frequent absence of viscid mucus would lead to the idea that it was more of a spasmodic nervous affection—a disease operating upon the subtissues; but from the fact that relief is only had after a free exudation of mucus, we are again brought to the conclusion that this membrane is most particularly at fault.

Nor is this view impaired in the least from the fact that asthma is frequently a sequel of many other diseases, as in rheumatism, pleurisy, cutaneous eruptions, or habitual discharges, as in hemorrhoids, or from a transfer of morbid action, for still it is found to attack this membrane. In a contra view, patients subject to it are, in some cases, instantaneously attacked from inhaling some noxious vapor. The atmosphere being impregnated with odorous essences, its occurrence in some fogs and mists, and its relief by this agency at other times, confirms the opinion that it must reside in the mucous membrane.

It is still more evident in the almost universal treatment prescribed. Squills, as a powerful expectorant, is also the most active stimulant of the mucous membrane, giving relief to the whole excernant system, by increasing the discharge of mucous; so with seneca root, a sort of general evacuent, but which always promotes the secretion of mucus, both being generally employed after the use of calomel or some other cathartic. Counter irritants, in this disease, act beneficially, by withdrawing the morbid influence from this membrane, until by the forementioned or other genial stimulants, its healthy secretion becomes restored, to the relief of the whole pulmonary apparatus.

We enter now upon a more common and better understood disease—gastritis; one in which the morbid effects upon the tissues of the mouth are recognized, not only by physicians and dentists, but by the patients themselves,—which has led to the error of referring almost all the diseases of the teeth too generally to this fruitful source. The immediate connection of the mouth and stomach is not of a closer intimacy than that subsisting between the mouth and the lungs, the bronchia, or in fact of any intervening part or passage whose surface is lined by the mucous membrane,—the only difference being in the greater extent and greater frequency of disturbed action; for it must be borne in mind that it is a membrane embracing, in various forms, all parts of the body, and from its great vascularity and large supply of the nervous filament, endowed with an unusual power of transmitting impressions to distant parts, and of sympathising with even remote organs, when suffering from disease.

In the buccal cavity, this condition of structure is still more exalted. The unceasing change of temperature, the perpetual contact of acrid and stimulating matter, the poisonous influence of miasmas, of whatever nature they may be, or whatever part of the system they may attack, produce their various morbid phenomena through the mucous membrane or its secretions. Indeed there can be little doubt but that the invisible agent, malaria, and even the poison communicating the various epidemics, as yellow fever, cholera, &c., accept of this agency to convey to the nervous system and blood its morbid influences, clothing and protecting as it does, by its peculiar fluid, all the vital organs of the system.

The stomach is a great centre of sympathy and of acute sensibility; it is hence impossible for inflammation to attack it without great suffering, and without extending its influence very widely. Gastritis is invariably accompanied by fever, and is generally characterized by an inflammatory color of the fauces, usually extending down the esophagus, and in many cases pervading the greater part of the alvine canal. The mucous membrane of the mouth is always affected in this disease; indeed, the surface and edges of the tongue give the most reliable indication of the state of the stomach, whenever any disturbance exists there; its middle and posterior surfaces are usually covered with a whitish fur during the first period, and in many cases before the disease has fully developed its character. Should the attack be slight, this appearance may remain unchanged; but when it becomes severe, the edges will be red, and sometimes swollen, while the papilla will protrude inflamed points through the furred surfaces, and the whole organ become dry and extended. When the disease becomes unfavorable to any treatment, the tongue throws off these peculiar conditions, and assumes a smooth red surface; is dry—becoming gradually covered with a thrush-like exudation; the skin becomes cool and pale; the mucous, which before had been ejected of a natural color, is now entirely changed to a blackish looking matter; the whole system sinks rapidly and death supervenes.

The chronic form of this disease is said by a celebrated writer to approach so gradually that it seldom attracts the serious attention of the patient, or comes before the notice of the physician until it has existed a considerable period; but during this time the mucous membrane is becoming more and more affected, with but little indication of this condition, except it is by a "sense of heat or burning, extending up the esophagus, over the chest," and the left side particularly, says the author before referred to. At first this is unattended by any eructations, but gradually, small quantities of gas, sometimes inodorous, at others offensive and irritating, are thrown off, accompanied by sour and acrimonious fluids. Frequently, the glairy, mucous secretions of the stomach and alvine canal are thrown up, along with the gas and fluid before mentioned. In the latter stages, the throat becomes excoriated, and not unfrequently blood is passed along with the food, bile, mucous, sour and acrid fluids, mixed it may be with these, or with the gastric juice.

In the meantime, the mouth is prominently affected, and treatment is always addressed to relieve the distress occasioned by it. In chronic gastritis the duration of the disease is such, and its progress so irregular, that patients often imagine themselves cured when a mere change of diet or sudden exposure will produce a recurrence; in fact, many inappreciable causes will arouse it to full force again, showing that its violence had only been subdued, and that it still lay dormant in the stomach. This state is characterized by derangement of the secretions, which empty upon or through mucous surfaces, especially those of the mouth; the saliva being found to be almost invariably acidulated; at times excessively abundant, even in some cases to salivation; at others it is found deficient in quantity, and still more acrid in quality, more particularly the fluids secreted by the sublingual and submaxillary, which partake more largely of this trouble than the parotids, which seem from their peculiar location, to be more exempt from disturbances arising from affections of the mucous membrane than the other large glands of the mouth. These glands, with the submucous, are always in a diseased condition when the stomach is in any way deranged, and consequently the gums are in an unhealthy state, incapable of giving that support and protection to the teeth that is essential to the continuance of their health.

Through this cause they frequently become loose and elongated, and sometimes drop out of their sockets; at others, becoming the seat of such severe pain and bodily discomfort as to imperatively demand their removal. I have known whole sets extracted from this cause. The irritation thus communicated to the mouth, gums, &c., seems to find an inordinate predisposition to inflammation in the periosteum, which, in these cases, terminates only in its entire destruction. I have often seen the most simple operations fail, being followed by the death of the organ, when performed during the existence of this disposition to inflammatory action in the dental periosteum, occasioned by gastritis; which operations, under a different condition of the system, would be unattended by any inconvenience or trouble.

The opinion that diseases take their allotted position in different parts of the body by some singular affinity or mysterious law of preference, seems to be peculiarly verified in this instance. Thus the stomach becomes attacked, and the mouth indicates it immediately by an inflammatory condition, retaining the morbid impressions not only during its existence in the primarily affected part, but long after it is the seat of any morbid action, and in fact appears as the immediate offspring of gastritis.

Dyspepsia is only another form of this disease, in which the irritation is of a very low order, and produced, not by any immediate action of some indigestible matter, or poisonous substances, but rather by continued depressing habits of body and mind, long indulged errors of diet, and by continued neglect of the premonitions of the appetite, as directed either to the amount taken, the time when, or of its analytical constituents, producing direct or indirect debility, generally by too great excitability from excessive stimulation. The mucous membrane is the first part to suffer; its villous coat becomes inflamed from the acrimony of the secretions, and from the inordinate and constant distention of the stomach by gaseous inflation, by the long detention of indigestible food, and by the perpetual efforts of the economy to overcome the evil, either by ejecting the matter, or by forcing it in an ill prepared manner into the vessels for distribution. The inflammation thus produced extends up the esophagus, as in gastritis, communicating this trouble to the mouth and its various integuments to such an extent, as in almost all cases to produce the decay of the teeth of such patients. Indeed, we may predict, with considerable certainty, their entire destruction.

It is not only from the consequences upon the mouth, as communicating actual disease to its glands and secretive structure, that the destruction of the teeth is attributable—but to other and no less formidable ones; the regurgitation of half digested food and chyme, in such an acidulated condition as to act as an immediate decomposer of dentine. This offensive discharge into the mouth is, with many patients suffering from dyspepsia, a very frequent, if not constant habit. Not unfrequently the larynx and fauces become so irritated as to induce a constant attempt to clear the throat by coughing. The mucous secretion is filmy and acrimonious, causing at times a sense of constriction exceedingly troublesome. The saliva is always altered in its quality and quantity; the mouth, after a disturbed sleep, being dry, furred and offensive, followed perhaps, in a few hours, by a discharge of a fluid of a very acrid nature. The mucous is frothy and tenacious, being ejected by considerable effort, and of a very sour and unpleasant taste. Indeed it would appear that this dreadful complaint locates its worst and most enduring consequences upon the teeth, from the fact that these organs are attacked at once, and continues its destructive influences long after it has ceased to exist in the stomach, from whence it originates. If of long duration, it will produce decay in teeth of the hardest and best formed structure, in spite of all the care that is generally bestowed upon the health and cleanliness of the mouth. Although it is often paroxysmal in its attacks, having intervals of apparently entire ease, yet, from its very origin to long after its perception by the patient, its effects can be traced by the peculiar condition of the mouth, by an experienced individual.

The gums will present at times a cadaverous appearance, becoming almost white, and upon being pressed will be found soft, and for an instant a deep red color will pervade the spot that has been pressed, the paleness returning at once. At times a deep blush can be noticed in the depressed portions, as if the gums were formed in stratas, and the second layer was inflamed, which was observed through the first by means of its thinness at these points. As the diseases increases its effects in the mucous membrane of the mouth, the apices of the gums between the teeth will become slightly inflamed, and present the appearance of brick dust, as is described by some in treating of phthisis pulmonalis; and further observation will, we opine, establish this symptom as one belonging peculiarly to dyspepsia, for it can always be seen in individuals where the disease is of long standing. The pain and regurgitations so constantly occurring after eating, are occasioned to a great extent by the food being taken into the stomach mixed with the diseased secretions of the mouth, which have become unfit to assist the gastric fluid in forming the chyme; instead of which there is found a mixture of various juices, with partly decomposed viands taken by a perverted appetite, which continue the almost ceaseless round of irritating influences, preventing every curative process of the economy.

Again, the morbid influence produced in the mouth by dyspepsia, is not effected alone by the direct action of the sour, bitter, acrid, oily and offensive matter thrown into it from the stomach, not only by the immediate communication of inflammation through the mucous membrane, but by the nervous irritability produced in the whole system. The derangement of almost all the sensations, the transitory but severe pains all over the body, in fact, the alteration of all physical and mental sensibilities evidence such a condition, and show how fully the nervous connection is sustained and effected, and also lends a positive proof of the disturbed state of all the secreting organs; these being regulated most powerfully by nervous influences. The mouth, therefore, and its many glandular appendages, must partake largely in the common disturbance, to the alteration of the fluids thereby produced, which cannot occur without injurious effects to the teeth. The nutrition of the whole system being equally at fault; the poisoned chyme must produce defective chyle, and this must again poison the blood, which becomes an irritant of the worst kind, as it pervades every fibre of the body, giving nourishment and secreting the various fluids of the glands, which are dependent upon its constituents for their chemical character.

In health this is found to present all that is required, and so abundantly as to give to these parts perhaps a greater recuperative power than is found in any other part of the body, so that we find ulceration of the mucous membrane lining the buccal pharyngeal parts capable of a very rapid regeneration, whilst this power is quite limited in the enteric and urinary. From these few facts, it is evident that a diseased condition of the blood originating in dyspepsia, must act as a morbid agent in the diseases of the gums, teeth, &c., it being the derivative source of all the fluids of the mouth, whether they come from the parotids, sublingual, submaxillary, or submucous glands; being diseased, the unavoidable conclusion is that they act as morbific agents upon the teeth and mouth generally.

Duodenitis and dysentery, in their effects upon the mouth, will be treated under the head of enteritis, as its name implies inflammation of the intestines; it pèrhaps will be better understood as one involving the mucous membrane lining the jejunum, ileum and colon, although the upper portions are generally considered most liable to inflammation, from their peculiar shape, position and structural arrangement, confining the fluids or other causes of irritation in this portion.

From what has been said of the power of communicating disease through the mucous membrane, it will be readily conceived that the diseases of the stomach often descend by this means to the part at present considered, and from the causes just enumerated, result in a secondary local affection of the jejunum, ileum, cœcum or colon. But as the object of this paper is to show that general disease, more particularly alimentary, affects the health of the mouth and ultimates in disease of the teeth, the causes producing the original disturbance in distant parts will necessarily be omitted, other than they may be found at times acting as morbific agents to both. Mr. Good, in his invaluable treatise on the practice of medicine, says, "that the sympathy of the mucous membrane of the intestines is extremely acute, and its sensibility very great." This is fully illustrated by the system at large suffering so extremely when it is inflamed; at such time the appetite fails, and an oppressive languor ensues, attended by general uneasiness; fever alternates with chilliness, the pulse varying accordingly; the skin is sometimes dry and again clammy; the tongue frequently moist, but as often dry and constantly furred. In fact, all the attending symptoms and consequences are so nearly allied to those of gastritis and dyspepsia, that we must refer to the matter written under these headings for further particulars, merely noticing that in fatal cases of enteritis, the lining membrane of the mouth generally ulcerates, and not unfrequently large portions of these surfaces slough away. The tongue becomes swollen, red, and finally assumes an aphthous condition, is covered by pustules resembling drops of candle wax. French authors give them the name of wax drops, in treating of this sign in *aphtha*, as a specific disease of the mucous membrane—of which we shall speak as we progress further in this subject. The fact that the mouth, in even a fatal termination of enteritis, becomes the seat of so dreadful a condition, is of itself sufficient to establish the tendency, in milder forms, to serious irritation in this cavity.

Indeed cases are recorded wherein the mucous membrane has been found, upon dissection, to have its entire surface—that is, its whole extent in the body, highly inflamed, even to an ulcerous condition of many parts, as before mentioned.

We are reminded at once, by referring to the fact that dental irritation is more frequent in the summer months, of the almost direct relationship existing between the stomach, lower intestines, mouth and teeth. Even a careless observer must have witnessed that as soon as fruit becomes a large portion of the diet of individuals, that the mouth assumes, almost simultaneously with the stomach and intestines, an irritated and inflamed condition; which condition resolves itself into gastritis or dysentery, without preventive means are adopted—leaving in its stead an irritable condition of the organs of secretion, especially the liver.

The treatment is not comprised in our province. We may state, however, that all food should partake of a mucilaginous character, of a nature to soothe the inflamed surfaces, and that when mercurial treatment is deemed expedient, its action must be carried until a strong impression upon the gums is made—and so sustained for a considerable time; showing again how closely connected with the health of the mouth and teeth is this class of disease—for its cure is often dependent upon the amount of absolute change produced in those secretions that are directly or indirectly connected with and dependent upon the condition of the mucous membrane.

(To be continued.)

